# Comprehensive Detection of Chromosomal and Genomic Abnormalities via Next-Generation Sequencing-Based Genomic Proximity Mapping Improves Diagnostic Classification of Hematologic Neoplasms

**DOI:** 10.3390/cancers17233775

**Published:** 2025-11-26

**Authors:** Xueyan Chen, He Fang, Yu Wu, Soheil Meshinchi, Kikkeri N. Naresh, Emily Reister, Kyle Langford, Stephen M. Eacker, Yajuan J. Liu

**Affiliations:** 1 Department of Laboratory Medicine and Pathology, University of Washington, Seattle, WA 98195, USAknaresh@fredhutch.org (K.N.N.); 2 Translational Science and Therapeutics Division, Fred Hutchinson Cancer Center, Seattle, WA 98109, USA; 3 Phase Genomics, Seattle, WA 98109, USA

**Keywords:** genomic proximity mapping, Hi-C sequencing, structural variants, copy number alterations, gene fusions, myeloid neoplasm, lymphoma

## Abstract

**Background/Objectives:** Accurate detection of all classes of genomic structural variants (SVs), including chromosomal rearrangements and copy number alterations (CNAs), is essential for the diagnosis and classification of hematologic neoplasms. Conventional cytogenetic methods currently serve as routine clinical tools for detecting SVs. However, each commonly used cytogenetic test has specific limitations, and sequential application of these different tests may delay timely diagnosis and treatment. **Methods**: In this study, we evaluated the feasibility and utility of genomic proximity mapping (GPM), a novel high-throughput chromosome conformation capture (Hi-C)-based next-generation sequencing (NGS) method, to identify chromosomal and genetic aberrations in hematologic neoplasms in the clinical setting. GPM was performed on 18 cases of hematologic neoplasms (fresh/frozen cells or formalin-fixed paraffin-embedded tissue), and concordance with other methodologies was assessed, including karyotyping, FISH, RT-PCR, chromosomal microarray analysis (CMA), and/or RNA sequencing. **Results:** GPM reliably detected balanced and unbalanced chromosomal rearrangements, including chimeric gene fusions and gene juxtapositions, with 95.2% concordance with previously applied methods in cases with >10% tumor burden. Additionally, GPM can detect CNAs and copy-neutral loss of heterozygosity (cnLOH) simultaneously in a single assay. Furthermore, detection of genomic rearrangements not identified by other methods improved the accuracy of disease classification. **Conclusions:** These findings demonstrate that GPM is a powerful method for identifying clinically actionable variants in hematologic neoplasms, overcoming some limitations of current cytogenetic technologies and improving the diagnostic accuracy and classification in challenging cases

## Simple Summary

Accurate diagnosis of blood cancers often depends on detecting chromosomal and genetic abnormalities. While current laboratory tests each have their strengths, they also have limitations, and multiple tests are often needed, which can delay diagnosis and treatment. In this study, we evaluated a novel method, genomic proximity mapping, which uses next-generation sequencing to detect a broad range of chromosomal changes in a single clinical assay. We applied this approach to cases of lymphoma and leukemia, including several that were difficult to classify using standard techniques. Genomic proximity mapping reliably identified both known and previously undetected abnormalities. In several cases, this led to more accurate classification. Our findings suggest that this method may improve diagnostic precision for blood cancers, allowing for faster and better-informed treatment decisions. We hope this technology will be adopted more widely in clinical practice and provide deeper insights into the pathogenesis of these diseases.

## 1. Introduction

Hematologic neoplasms, including myeloid and lymphoid neoplasms, frequently harbor diverse genomic structural variants (SVs), such as chromosomal rearrangements and copy number alterations (CNAs). The updated diagnostic criteria for various hematologic neoplasms, as outlined in the 5th edition WHO classification and the 2022 International Consensus Classification (ICC), have expanded the diagnostic framework by incorporating an extensive list of genomic aberrations in addition to morphologic and immunophenotypic features [1,2]. In myeloid and lymphoid neoplasms, a subset of recurrent chromosomal rearrangements results in gene fusions that either encode chimeric proteins or reposition genes under the influence of different regulatory elements, which in turn drive disease pathogenesis. Many of these rearrangements are recognized as disease-defining aberrations. Therefore, timely and accurate detection of all types of SVs is critical for definitive classification, risk stratification, and optimal management of hematologic neoplasms.

Conventional cytogenetic techniques, such as chromosome analysis, fluorescence in situ hybridization (FISH), and chromosomal microarray analysis (CMA), as well as short-read next-generation sequencing (NGS), currently serve as the routine laboratory tools to detect chromosomal rearrangements and CNAs. However, each of these standard technologies has specific limitations, including limited resolution leading to imprecise breakpoint mapping, inability to detect balanced rearrangements, and difficulty detecting cryptic rearrangements. FISH is limited by the requirement for multiple probe sets, which increases both cost and turnaround time. Moreover, FISH cannot practically identify all potential fusion partners or accurately determine exact breakpoints. Routine clinical application of whole-genome NGS methods could provide a solution but is also challenging to detect somatic variants due to limited read length and cost to achieve adequate sequencing depth [3,4]. Long-read sequencing technologies show promise for the comprehensive discovery of SVs; however, achieving high performance in clinical settings remains challenging due to a lack of mature computational analysis tools and high sequencing costs [5,6,7]. These limitations highlight the need for developing novel high-throughput technologies that provide better resolution, more comprehensive characterization, reduced reliance on reflex testing, and potential for new discoveries.

Recent studies have demonstrated the applicability of newly emerged, DNA-based high-throughput chromosome conformation capture (Hi-C) NGS technology for detecting SVs including balanced and unbalanced chromosomal rearrangements and CNAs in hematologic malignancies, solid tumors, and constitutional disorders [8,9,10,11,12,13]. In this study, we evaluated the clinical utility of one such Hi-C-based method, genomic proximity mapping (GPM) [14,15], for the comprehensive characterization of chromosomal aberrations in challenging hematologic neoplasms to improve diagnostic accuracy and provide insights into disease pathogenesis.

## 2. Materials and Methods

### 2.1. Case Selection

To evaluate the performance of GPM in detecting various types of genomic aberrations, including chromosomal translocations, inversions, insertions, deletions, duplications, and/or cnLOH, 18 cases encompassing a variety of hematologic neoplasms were selected by searching the University of Washington Pathology database from 2020 to 2023. Most of these cases had known chromosomal rearrangements or CNAs detected by chromosome analysis, FISH studies, reverse transcriptase (RT)-PCR, RNA sequencing, and/or CMA, such as cases with known gene juxtapositions and chimeric gene fusions. The pathologic diagnoses included various types of mature lymphomas and myeloid neoplasms. Additionally, five normal peripheral blood samples were selected as negative controls. One standard cell line (HG002) was used as a positive control [16]. The sample types consisted of fresh or frozen cells from peripheral blood and bone marrow aspirate (12 cases) and FFPE lymph node or other neoplasm-involved tissue (6 cases). Clinical and demographic characteristics, pathologic diagnoses, flow cytometry, immunohistochemistry, and cytogenetic findings were reviewed. Panel-based NGS was performed in a subset of cases as routine clinical testing. This study was approved by the University of Washington Institutional Review Board.

### 2.2. Genomic Proximity Mapping

Hi-C library preparation for GPM was conducted with a prototype version of the Phase Genomics CytoTerra kit. For FFPE samples, three 5 µm or one 15 µm curls were used as input for chromatin extraction using Phase Genomics protocols and a Covaris S220 adaptive focused acoustics. For cellular samples, between 200,000 and 500,000 cells were crosslinked using 1% formaldehyde and used as input. Following lysis, chromatin was immobilized on magnetic chromatin recovery beads and digested by restriction enzymes. The resulting overhang ends were filled in with biotinylated nucleotides and subjected to proximity ligation. Crosslinks were then enzymatically reversed and proximity ligated DNA was purified and enriched using streptavidin magnetic beads. Streptavidin-bound DNA was used to generate an Illumina-compatible paired-end sequencing library. The libraries were sequenced on an Illumina NovaSeq 6000 in a 2 × 150 bp configuration with a targeted read depth of 150 M read pairs per sample. FASTQ files were uploaded to the Phase Genomics CytoTerra platform for analysis. In brief, reads were mapped to the genome using BWA using the Hi-C flags [17]. Duplicate reads were marked using Samblaster and duplicates were removed using a custom-developed package, Matlock. Small variants were predicted using a convolutional neural network (CNN) and resulting variants were used for minor allele frequency (MAF) measurements. Large structural variants were evaluated using an ensemble of tools which include a proprietary CNN that operates on the contact matrix generated by the Pairix v0.3.7 [18] and Cooler v0.10.2 packages [19], an SVM that measures aneuploidy based on MAF, a specialized bioinformatic tool designed to identify breakpoints, and a trio of bioinformatic tools designed to detect CNAs and cnLOH using coverage and MAF information. Collectively, this ensemble of tools is referred to as the CytoTerra Caller Suite (v1.1). Following variant calling, all calls were manually inspected using the CytoTerra Curator tool for call visualization.

## 3. Results

### 3.1. Patient Characteristics (Table 1)

The evaluation cohort comprised 18 cases with diagnoses of various hematologic neoplasms, including mature T-cell and B-cell lymphomas, acute lymphoblastic leukemia/lymphoma, acute myeloid leukemia (AML), and other myeloid neoplasms, as detailed in Table 1. The analyzed tissue types included 12 fresh or frozen cell samples from peripheral blood and bone marrow, and six FFPE samples from bone marrow or lymph nodes. The proportion of neoplastic populations identified by flow cytometry varied widely, ranging from 0.75% to 86%, with one third of cases demonstrating fewer than 20% abnormal cells.

**Table 1:** Patient characteristics of case cohort.

	Number (%)
Patients No.	18
Age, median (range)	57 (16–82)
Gender	
Male	9 (50%)
Female	9 (50%)
Tissue type	
Peripheral blood	6 (33.3%)
Bone marrow	7 (38.9%)
Lymph node	5 (27.8%)
Pathologic diagnosis	
Acute lymphoblastic leukemia	4 (22.2%)
Acute myeloid leukemia	3 (16.7%)
Diffuse large B-cell lymphoma	3 (16.7%)
High-grade B-cell lymphoma	2 (11.1%)
Mantle cell lymphoma	1 (5.6%)
T-prolymphocytic leukemia	1 (5.6%)
Primary myelofibrosis	1 (5.6%)
Myelodysplastic neoplasm	1 (5.6%)
Chronic myeloid leukemia in B-lymphoid blast crisis	1 (5.6%)
Rosai–Dorfman disease	1 (5.6%)
Abnormal cells by flow cytometry, median (range)	0.75–86%
<20%	7.9% (0.75–15.8%)
20–50%	38.0% (29.0–49.3%)
>50%	75.0% (60.2–86.0%)

### 3.2. Comparison of Genomic Aberrations Detected by GPM and Other Cytogenomic Methods, Including Novel Findings by GPM (Table 2)

**Figure 1:**
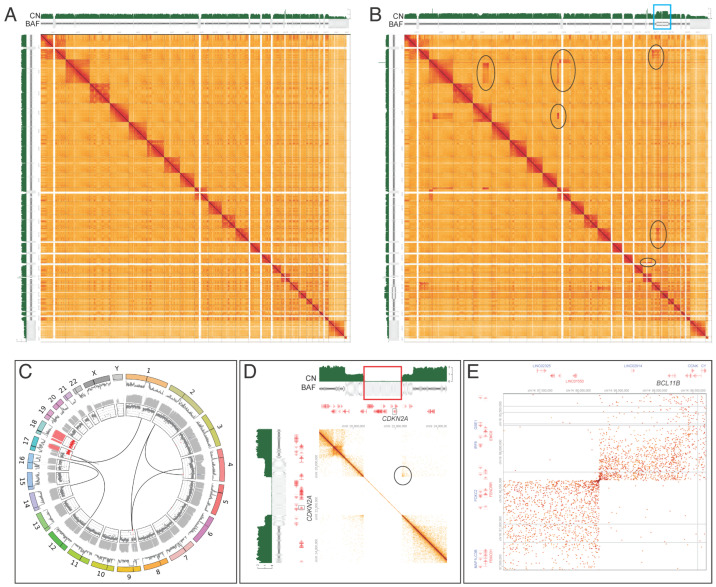
Whole-genome view highlighting structural variants (SVs) and copy number alterations (CNAs) detected by GPM. Two-dimensional (2D) contact heatmaps are shown to visualize chromosomal interactions across the genome (1-22,X,Y), symmetrical along the diagonal line. (**A**) A representative normal sample shows typical chromatin interaction patterns. (**B**) An abnormal sample (case 11) is characterized by multiple chromosomal rearrangements (black circles) and CNAs, such as copy gain of 17q and 18 (blue box). (**C**) Circos plot represents multiple chromosomal rearrangements and CNAs detected in case 11. Rings represent, from outer to inner, chromosomes (lines indicate centromere location), raw read depth, GC-corrected coverage, and B-allele frequency (copy gain of 17q and 18 colored red). Lines within the rings indicate location of intra- and interchromosomal SV breakpoints. (**D**) *CDKN2A* deletions include a biallelic deletion (red box) resulting in contact by discontinuity (CBD) showing in the black circle. (**E**) Cryptic t(14;16) translocation involving *BCL11B* is detected.

Of the 15 cases harboring specific genetic aberrations identified by karyotype, FISH, CMA, and/or RNA sequencing, GPM detected genetic aberrations in 14 cases. Hi-C data used for GPM are commonly visualized as a two-dimensional heatmap that represents pairwise sequence interactions, as illustrated in Figure 1, including a normal sample (Figure 1A) and an abnormal sample (Figure 1B, case 11) with multiple rearrangements and CNAs (Figure 1C–E). The frequency of pairwise interactions is determined by the linear distance of sequences along the chromosome, such that sequences that are closer interact at a higher frequency [20]. Testing on the standard normal cell line (HG002) revealed expected results and no abnormalities were detected in five normal peripheral blood samples [16]. Remarkably, GPM uncovered abnormalities in two of the three cases that conventional assays had classified as normal. A Rosai–Dorfman disease case (case 18) remained without any detectable aberrations.

**Table 2:** Comparison of GPM and genetic results in the diagnosis of leukemias and lymphomas.

Case	Original Pathologic Diagnosis	Diagnostic Impact of GPM	GPM (Relevant Aberrations)	Karyotype/FISH
1	Mantle cell lymphoma	Comprehensive genomic analysis	t(11;14) *IGH::CCND1*, *TP53* deletion, other CNAs	nuc ish(CCND1,IGH)x3(IGH con CCND1)x2[188/200]
2	High-grade B-cell lymphoma	Diagnostic support for Burkitt lymphoma	t(8;22) *IGL::MYC*	nuc ish(BCL6)x2[200],(MYC)x2(5′MYC sep 3′MYC)x1[200],(MYCx3,IGHx2)[200],(IGH,BCL2)x2[200]
3	High-grade B-cell lymphoma	Comprehensive genomic analysis	6q deletion, 9p cnLOH, copy gains of segments of 2p, 11p, and 12q, +13, +17, +22	nuc ish(MYC)x2[200]
4	Diffuse large B-cell lymphoma	Accurate genetic characterization for diagnostic clarity	t(12;22) *IGL::CCND2*, inv(3) *RPN1::EVI1*, t(5;15), t(10;18)	46,XY,add(3)(q27)[7]/46,XY,t(5;15)(q33;q15),t(10;18)(p10;p10),t(12;22)(p13;q11.2)[13]. nuc ish(CCND1,IGH)x2[200],(TP53,CEP17)x2[200]
5	Diffuse large B-cell lymphoma transformed from follicular lymphoma	Diagnostic support for DLBCL/high-grade B-cell lymphoma with *MYC* and *BCL2* rearrangements	t(8;9) *PAX5::MYC*, t(14;18) *IGH::BCL2*, 11q aberration, *TP53* deletion, other CNAs	nuc ish(5′MYCx2~3,3′MYCx1~2)(5′MYC con 3′MYC)x1~2[152/300],(IGH,BCL2)x2~3(IGH con BCL2)x1~2[171/200] *PAX5::MYC* (Exon2::Exon2) by RNA sequencing
6	Diffuse large B-cell lymphoma	Comprehensive genomic analysis	Multiple CNAs	nuc ish(MYC)x2[200]
7	Peripheral T-cell lymphoma	Diagnostic support for T-prolymphocytic leukemia	ins(14;14)(q11.2;q32.1q32.2) *TRA/D::TCL1A*	nuc ish(TCL1Ax3,TCL1Bx2)(TCL1A con TCL1B)x2[138/200]
8	B-lymphoblastic leukemia/lymphoma	Comprehensive genomic analysis	t(9;22) *BCR::ABL1*, 7p deletion (*IKZF1*), 17q deletion	46,XX,t(9;22)(q34;q11.2)[13]/46,sl,der(16)t(1;16)(q12;q11.2)[2]/47,sl,-16,+2r [5]. nuc ish(ABL1x3,ASS1x2,BCRx3)(ABL1 con BCR)x2[177/200] *BCR::ABL1* p190 by RT-PCR
9	B-lymphoblastic leukemia/lymphoma	Comprehensive genomic analysis	t(4;11) *AFF1::KMT2A*, trisomy 6, 7q gain	46,XY[20]. nuc ish(KMT2A)x2(5′KMT2A sep 3′KMT2A)x1[181/200]
10	B-lymphoblastic leukemia/lymphoma	Accurate genetic characterization for diagnostic clarity	t(5;14) *IGH::IL3*, t(4;15) *BMP2K::SMAD3*	t(5;14) (per outside report)
11	T-lymphoblastic leukemia/lymphoma	Accurate genetic characterization for diagnostic clarity	t(1;17), der(12)t(12;17), t(2;9;4), t(14;16) with *BCL11B* rearrangement, biallelic CDKN2A deletion, +18	47,XY,t(1;17)(q21;p13),t(2;9;4)(p23;p23;q25),del(4)(q27q31),der(12)t(12;17)(p13;q11.2),+18[33]/47,XY,idem,add(4)(q35)[5]/47,XY,idem,del(4)(q11.2q25)[2]. nuc ish (CDKN2Ax0,CEP9x2)[194/200]
12	Acute myeloid leukemia	Comprehensive genomic analysis	inv(16) *MYH11::CBFB*, t(1;12), t(6;12)	46,XY,inv(16)(p13.1q22)[13]/46,idem,inv(7)(p13p22)[5]/46,idem,t(1;12)(q32;p13)[2]
13	Acute myeloid leukemia	Comprehensive genomic analysis	Deletions of 3p and 5q, and other CNAs	52,XX,del(3)(p13),del(5)(q13q33),+del(5)(q13q33),add(9)(q34),+11,+13,+14,+21,+22[8]/52,sl,add(14)(q32)[cp6]/45~48,del(3)(p13),t(4;11)(q25;q25),del(5) (q13q33),add(9)(q34),add(12)(p13)[cp3]/46,XX,-3,del(5)(q13q33),der(12)t(3;12)(q21;p13),-18,add(21)(q22),+2mar [cp3]. nuc ish(D5S23x2,EGR1x1)[50/200]/(D5S23x3,EGR1x1)[68/200]
14	Acute myeloid leukemia	Concordant	Normal	46,XX[20]
15	Chronic myeloid leukemia in B-lymphoid blast crisis	Concordant	t(9;22) *BCR::ABL1*	t(9;22) by FISH (per outside report) Negative for *BCR::ABL1* by RT-PCR *BCR::ABL1* (Exon 13::Exon 3) by RNA sequencing
16	Primary myelofibrosis	Concordant	9p cnLOH	46,XX[20]
17	Myelodysplastic neoplasm	Comprehensive genomic analysis	Deletions of 5q, 16q, 17p, and 17q, -18, der(20)t(5;20), and other CNAs	43,X,-Y,-5,-17,-18,add(20)(p13),+r[6]/44,idem,+r[10]/43,idem,-r,+mar[2]/46,XY[2]. nuc ish(EGR1x1,D5S23x2)[184/200]
18	Rosai–Dorfman disease	Concordant	Normal	Failed

#### 3.2.1. Mature Lymphomas

In a case of mantle cell lymphoma (case 1), GPM identified the expected t(11;14) *IGH::CCND1*, demonstrating concordance with the results of FISH studies and confirming the pathologic diagnosis. In addition, GPM detected complex genomic abnormalities, including TP53 deletion (Table 2).

**Figure 2:**
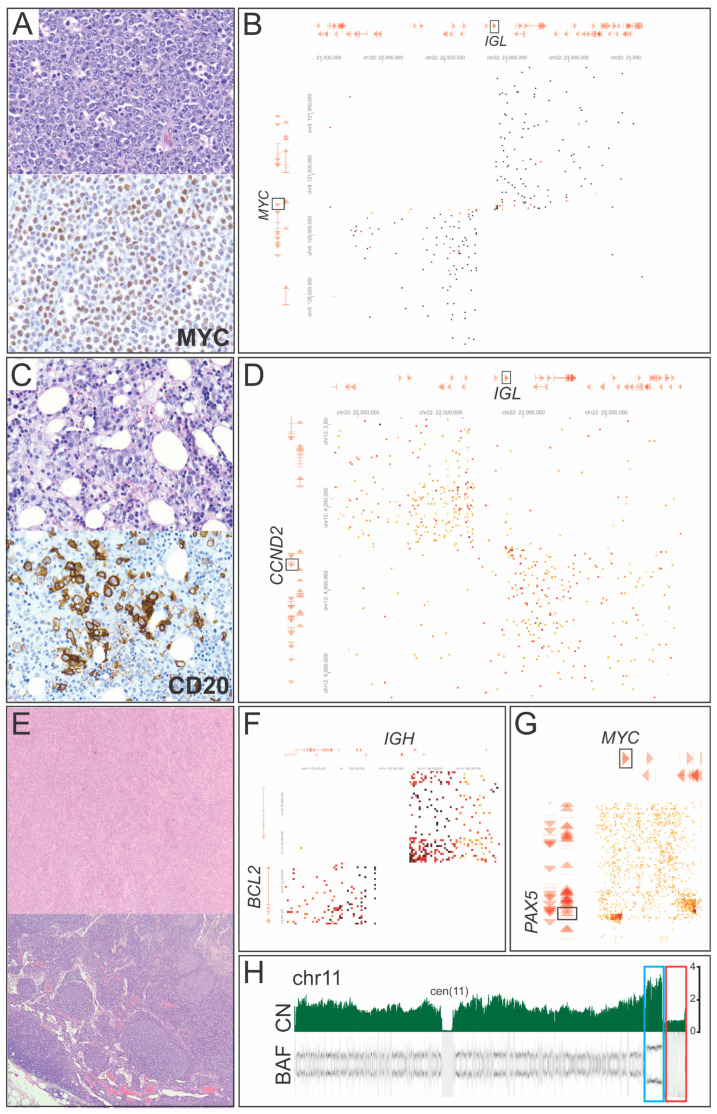
GPM detected gene rearrangements in various types of lymphomas including enhancer/promoter hijacking and chimeric gene fusions. (**A**,**B**) Case 2 with an original pathologic diagnosis of high-grade B-cell lymphoma, favoring Burkitt lymphoma. H&E-stained section of the lymph node biopsy shows a diffuse infiltrate of large atypical B-cells with co-expression of *MYC* by immunohistochemistry (**A**) and GPM detected t(8;22) *IGL::MYC* fusion (**B**). (**C**,**D**) Case 4 with an original pathologic diagnosis of bone marrow involvement by large B-cell lymphoma. H&E-stained section of the bone marrow biopsy shows an infiltrate of large B-cells that are positive for CD20 by immunohistochemistry (**C**). GPM detected t(12;22) *IGL::CCND2* fusion (**D**). (**E**–**H**) Case 5 with an original pathologic diagnosis of large B-cell lymphoma. H&E-stained sections of the sample demonstrate involvement by a large B-cell lymphoma (upper) in the background of a FL (lower) (**E**). GPM identified t(14;18) *IGH::BCL2* (**F**) and t(8;9) *PAX5::MYC* fusions (**G**). GPM also identified aberrations of chromosome 11 including a copy gain of most of chromosome 11 and 2-copy gain of 11q24.1-q24.2 (blue box) with terminal loss of 11q24.2-qter (red box) (**H**).

Case 2 was diagnosed with high-grade B-cell lymphoma, with phenotypic features favoring Burkitt lymphoma, despite exhibiting greater cytomorphologic variation (Figure 2A). FISH revealed *MYC* rearrangement, but the partner gene was not *IGH*, the typical fusion seen in Burkitt lymphoma. GPM identified *IGL::MYC* (Figure 2B), which is observed in 10–20% of Burkitt lymphoma cases, providing further support for a diagnosis of Burkitt lymphoma.

Case 3 revealed B-cell lymphoma with Burkitt lymphoma-like morphologic and immunophenotypic features. FISH studies were negative for *MYC* rearrangement. GPM likewise did not identify any disease-defining genetic aberrations, but did reveal multiple CNAs, including 6q deletion, 9p cnLOH, copy gains of segments of 2p, 11p, and 12q, +13, +17, +22, indicating complex genomic aberrations. The combined findings argue against a diagnosis of Burkitt lymphoma and are more consistent with high-grade B-cell lymphoma, not otherwise specified (NOS).

Case 4 demonstrated a diffuse large B-cell lymphoma (DLBCL) involving the bone marrow, in the setting of a prior diagnosis of chronic lymphocytic leukemia/small lymphocytic lymphoma (CLL/SLL) (Figure 2C). Chromosomal analysis revealed a complex karyotype, including t(12;22), t(5;15), and t(10;18) in one clone, and add(3) in a separate clone. FISH studies were negative for t(11;14) *IGH::CCND1*, and *TP53* deletion. GPM identified t(12;22) *IGL::CCND2* (Figure 2D), and inv(3) *RPN1::MECOM*, and other SVs and CNAs concordant with the karyotype. A whole-arm reciprocal translocation between chromosomes 10 and 18 was also visible by the excess signal observed on the heatmap.

Case 5 had a history of follicular lymphoma (FL), and the pathologic sample demonstrated a composite morphology, containing both FL and DLBCL (Figure 2E). FISH revealed a separate signal at the 5′ end of the *MYC* gene without a definitive rearrangement, as well as t(14;18) *IGH::BCL2* fusion present in the majority of cells, consistent with transformation from FL. RNA sequencing revealed an inframe *PAX5::MYC* (Exon2::Exon2) fusion but was unable to detect the *IGH::BCL2* fusion as an inherent limitation of this technology. CMA identified numerous CNAs, including chromosome 11q gain with telomeric loss. GPM confirmed the presence of both *IGH::BCL2* (Figure 2F) and *PAX5::MYC* (Figure 2G), along with 11q aberrations (Figure 2H) and other complex genomic abnormalities, supporting a diagnosis of DLBCL/high-grade B-cell lymphoma with *MYC* and *BCL2* rearrangements.

Case 6 was diagnosed with DLBCL, germinal center B-cell subtype by the Hans algorithm [21]. The lymphoma exhibited a Burkitt lymphoma-like immunophenotype and a high proliferation rate of ~90%, but FISH was negative for *MYC* rearrangement. GPM identified complex genomic aberrations but did not detect any disease-defining fusions or specific genomic aberrations.

**Figure 3:**
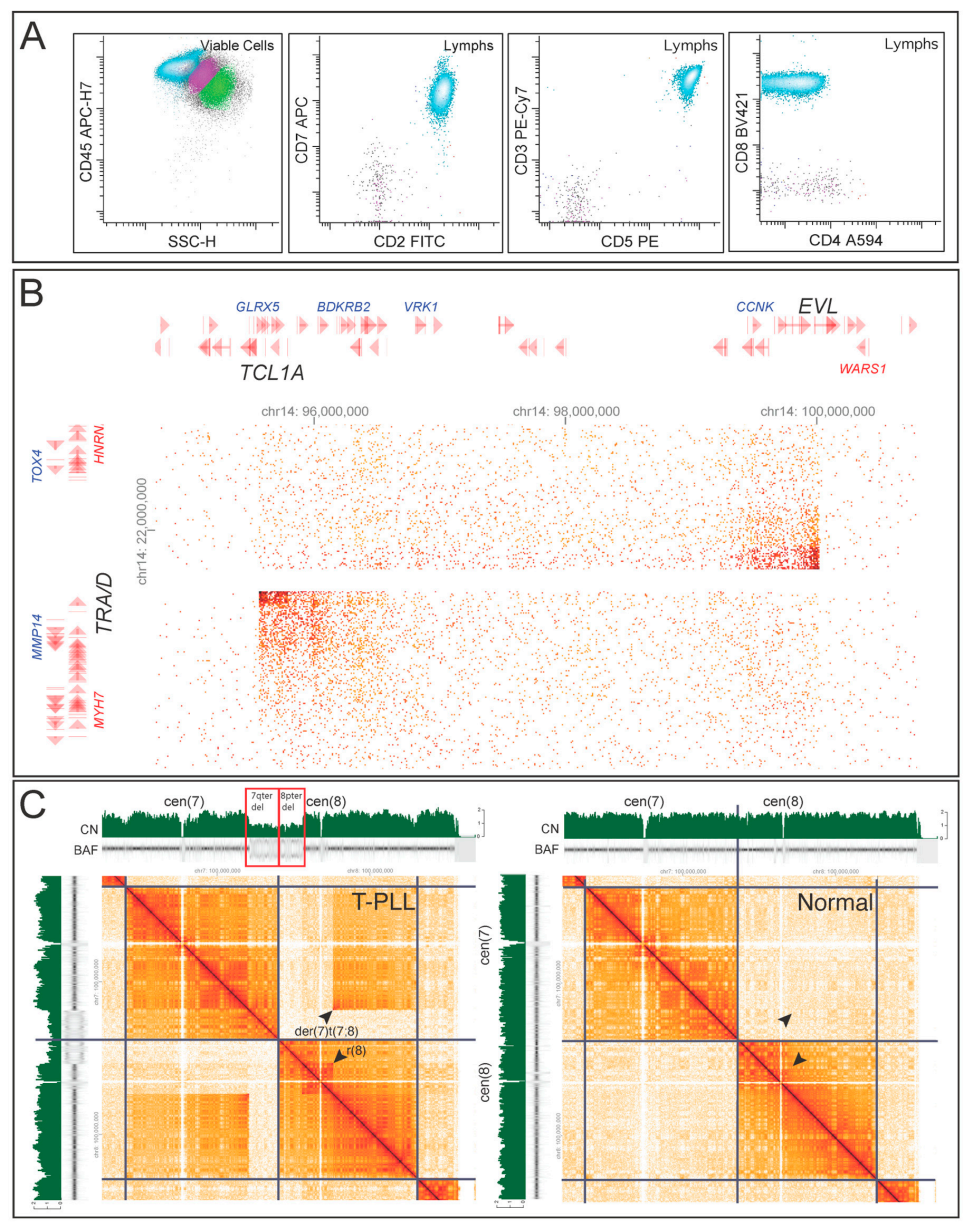
GPM detected *TRA/D::TCL1A* fusion and complex genomic aberrations, confirming the diagnosis of T-PLL (case 7). (**A**) Flow cytometry of peripheral blood revealed an abnormal T-cell population (colored aqua) expressing CD2, CD3, CD5, CD7, and CD8 without CD4. Monocytes are colored pink and granulocytes green. (**B**) GPM identified an inverted insertion of the 14q32.13–q32.2 fragment containing *TCL1A* into *TRA/D* at 14q11.2, resulting in a *TRA/D::TCL1A* fusion. (**C**) Compared to a normal sample (right panel), GPM revealed complex genomic alterations, including a derivative chromosome 7 with 8q12.1-qter translocated to 7q32.1 (arrowhead), resulting in a 7q32.1-qter deletion (red box), and a ring chromosome 8 formed by the junction of 8p21.1 and 8q12.1 (arrowhead), accompanied by an 8p21.1-pter deletion (red box).

Case 7 showed an abnormal T-cell population in the peripheral blood by flow cytometry (Figure 3A), consistent with T-cell leukemia/lymphoma that could not be further subclassified based on the immunophenotype. FISH revealed an extra signal at *TCL1A*; it was unclear whether this represented a rearrangement or trisomy. GPM confirmed an inverted insertion of the 14q32.13-q32.2 fragment containing *TCL1A* into *TRA/D* at 14q11.2, resulting in *TRA/D::TCL1A* fusion (Figure 3B), consistent with a diagnosis of T-prolymphocytic leukemia (T-PLL). GPM also identified additional complex genomic aberrations, including a derivative chromosome 7 with t(7;8) translocation and a ring chromosome 8 (Figure 3C).

#### 3.2.2. Acute Lymphoblastic Leukemia/Lymphoma

**Figure 4:**
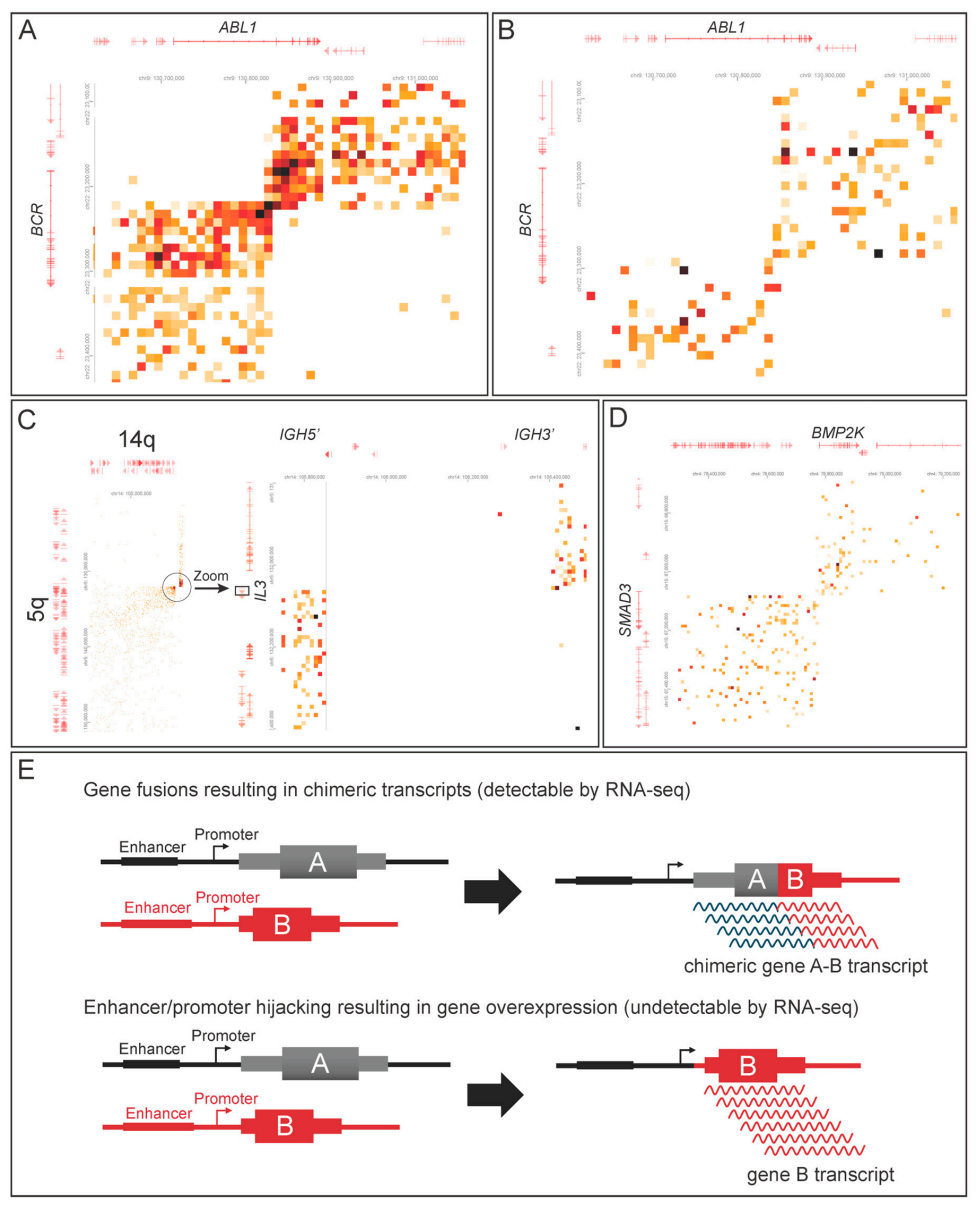
GPM detects two major types of gene rearrangements including gene fusions with exon-level resolution and enhancer/promoter hijacking. (**A**) GPM identified a t(9;22) *BCR::ABL1* fusion involving *BCR* exon 1 and *ABL1* exon 2 in a case of B-LL (case 8). (**B**) A t(9;22) *BCR::ABL1* fusion involving *BCR* exon 13 and *ABL1* exon 3 was detected in CML (case 15), revealing an atypical breakpoint for *ABL1*. (**C**) In a case of B-LL (case 10), GPM detected t(5;14) *IGH::IL3* fusion and, (**D**) a novel t(4;15) *BMP2K::SMAD3* fusion. (**E**) A diagram illustrates the mechanism of two types of gene rearrangements, including gene fusions producing chimeric transcripts, and enhancer/promoter hijacking causing gene overexpression.

Three B-lymphoblastic leukemia/lymphoma (B-LL) cases harbored disease-defining fusions by chromosomal analysis and/or FISH studies. In case 8, GPM accurately detected *BCR::ABL1* fusion involving *BCR* exon 1 and *ABL1* exon 2 (Figure 4A), illustrating its ability to resolve exon-level breakpoints resulting in chimeric transcripts (Figure 4E). GPM also identified CNAs in this case, concordant with CMA findings. In case 9, GPM confirmed *KMT2A* rearrangement detected by FISH and identified *AFF1* as the fusion partner. Chromosomal analysis showed a normal karyotype, likely due to the markedly hemodilute bone marrow aspirate contributing to the false negative results in addition to culture bias that can occur in acute lymphoblastic leukemia [22]. Case 10 revealed t(5;14) translocation by chromosomal analysis. GPM not only recognized it but also identified the specific fusion partner, *IGH::IL3* (Figure 4C), representing enhancer/promoter hijacking leading to *IL3* overexpression (Figure 4E). Additionally, a novel fusion t(4;15) *BMP2K::SMAD3* was identified by GPM (Figure 4D). The clinical significance of this variant has not been estalished in B-LL, but its detection shows the potential of GPM to uncover previously unrecognized genomic alterations.

Case 11 showed abnormal T-lymphoblasts in the peripheral blood, diagnostic of T-lymphoblastic leukemia/lymphoma (T-LL). Chromosomal analysis revealed a complex karyotype including multiple translocations involving chromosomes 1, 2, 4, 9, 12, and 17. CMA and FISH also identified biallelic deletion of *CDKN2A*. GPM confirmed these translocations (Figure 1C) and the biallelic *CDKN2A* deletion (Figure 1D). Most significantly, GPM identified a cryptic translocation t(14;16) resulting in *BCL11B* rearrangement (Figure 1E), which defines a heterogeneous group of acute leukemias [23] and was not detected by chromosomal analysis.

#### 3.2.3. Acute Myeloid Leukemia

Three cases of AML (cases 12–14) were included in this evaluation cohort. In case 12, chromosomal analysis revealed inv(16), inv(7), and t(1;12) translocation. GPM confirmed inv(16) *MYH11::CBFB*, and t(1;12), but failed to detect inv(7). Additionally, GPM identified a previously unrecognized t(6;12) translocation, with unknown clinical significance. In case 13, chromosomal analysis showed a complex karyotype, including deletions of 3p and 5q, and GPM confirmed these abnormalities. Case 14 had a normal karyotype, and CMA identified an intragenic deletion of *RUNX1* (~25 kb) in chromosome 21, concordant with a high variant allele frequency (91%) of a *RUNX1* mutation detected by NGS myeloid gene panel. However, GPM showed a normal result and did not identify *RUNX1* deletion.

#### 3.2.4. Other Myeloid Neoplasms

Case 15 was diagnosed with chronic myeloid leukemia (CML) in B-lymphoid blast crisis. Chromosomal analysis was not informative due to limited cell growth. FISH revealed *BCR::ABL1* fusion, whereas RT-PCR did not detect *BCR::ABL1* p190 or p210 transcripts. RNA sequencing identified *BCR::ABL1* fusion (Exon 13::Exon 3), revealing an atypical *ABL1* breakpoint. GPM confirmed *BCR::ABL1* fusion at the exon level (Figure 4B), and no other clinically significant aberrations were identified.

Case 16 had a diagnosis of primary myelofibrosis (PMF), with a normal karyotype. CMA showed 9p cnLOH in 90% of the cells, concordant with the high variant allele frequency of *JAK2 V617F* mutation. GPM confirmed 9p cnLOH.

In case 17 with myelodysplastic neoplasm(MDS), chromosomal analysis revealed multiple monosomies, including chromosomes 5, 17, and 18. FISH and CMA confirmed 5q deletion, 17p and 17q deletions without *TP53* deletion, and monosomy 18. GPM results were concordant with these findings and additionally identified a derivative chromosome 20 with t(5;20) translocation and an inv(17) of uncertain clinical significance.

### 3.3. GPM Performance and Concordance with Other Methods (Table 3)

**Figure 5:**
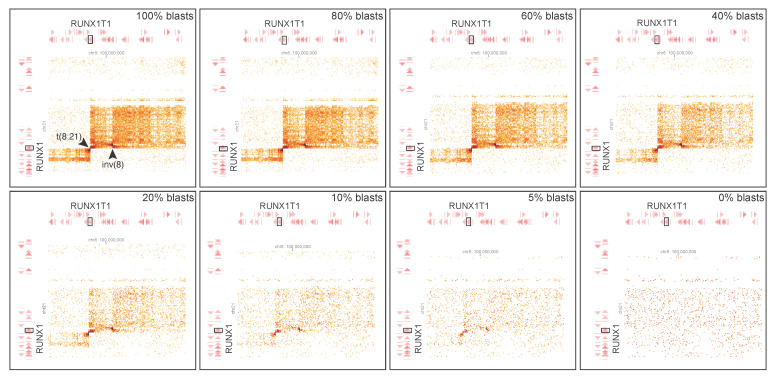
Serial dilution test determined the limit of detection of t(8;21) *RUNX1::RUNX1T1* (arrowhead) with an inversion 8q (arrowhead) by GPM. Serially in silico diluted specimens contained 100%, 80%, 60%, 40%, 20%, 10%, 5%, and 0% leukemic reads, respectively.

To determine the limit of detection of GPM, dilution tests were performed using varying proportions of an AML sample harboring t(8;21) *RUNX1::RUNX1T1* and an inversion 8q, admixed with matched GPM library derived from remission bone marrow cells. The percentage of leukemic reads mixed in silico with matched remission reads were 100%, 80%, 60%, 40%, 20%, 10%, 5%, and 0%. The limit of detection for the translocation and inversion by CytoTerra variant callers was confidently established at 10% leukemic blasts, although these aberrations were still visually detectable at 5% (Figure 5).

**Table 3:** Characteristics of GPM performance. (**A**) GPM performance on detecting structural variants. (**B**) GPM performance on detecting copy number alterations and cnLOH.

**(A)**					
	**Total**		**Translocation**	**Inversion**	**CBD**
Total variants by GPM	91		60	14	17
Variants by other tests *	31		21	3	7
Additional variants by GPM	62		40	12	10
Concordant variants by GPM	29		20	2	7
Concordance rate (%)	93.5		95.2		
**(B)**					
	**Total**	**Deletion**	**Duplication**	**Aneuploidy**	**cnLOH**
Variants by other tests *	83	32	18	11	22
Concordant variants by GPM	74	31	18	10	15
Concordance rate (%)	89.2	96.9	100	90.9	68.2

* Other tests include chromosomal analysis, FISH studies, CMA, and/or RNA-sequencing. CBD, Contact by discontinuity; cnLOH, copy neutral loss of heterozygosity.

Among all 18 cases, GPM detected a total of 91 SVs, including translocations, inversions, and contact by discontinuity (CBD). Based on previous clinical testing including chromosomal analysis, FISH, CMA, and/or RNA sequencing, 31 SVs were identified prior to GPM testing (Table 3A). Of these, 29 SVs were concordantly detected by both GPM and routine cytogenomic methods, yielding an overall concordant rate of 93.5%. One unbalanced translocation der(16)t(1;16)(q12;q11.2) (case 8) was detected by chromosomal analysis but not identified by GPM, resulting in a concordance rate of 95.2% for translocation detection. GPM also identified 62 additional SVs that had not been identified by other methods; these SVs were not included in concordance rate calculations. The size of all 14 inversions detected by GPM was >2.5 Mb, and 17 CBD > 1.3 Mb in this cohort.

Similarly, compared to CNAs and cnLOH detected by CMA in nine cases, the concordance rates between GPM and CMA for detecting deletions, duplications, aneuploidy, and cnLOH were 96.9%, 100%, 90.9%, and 68.2%, respectively (Table 3B). The size of all deletions detected by GPM was >100 kb, duplications >750 kb, and cnLOH > 1 Mb in this cohort. GPM failed to detect a *RUNX1* deletion (~25 kb, case 14), one aneuploidy (case 13) present in a low-level subclone, and seven cnLOH (31.8%) that had been recognized by CMA.

## 4. Discussion

### 4.1. Performance of GPM

GPM is a whole-genome, short-read sequencing method that retains three-dimensional chromosomal structure conformation, allowing for comprehensive characterization of genomic structure including SVs, CNAs, and cnLOH. As a genome-wide assay, GPM does not require cell culture and is therefore not affected by culture biases, a limitation that reduces the diagnostic yield of karyotyping in ALL [22]. Unlike FISH or targeted RNA sequencing, GPM is not restricted to specific targets, increasing the likelihood of identifying cryptic rearrangements and novel genomic aberrations. Other cytogenomic methods, such as optical genome mapping (OGM) and long-read sequencing (LRS), require high-quality, ultra-high molecular weight DNA, which limits their applicability in routine diagnostic testing; therefore, FFPE samples are not suitable for these methods. In contrast, GPM can be performed using fresh cells, fixed cell pellets, and FFPE samples from a wide range of tissue types. Its compatibility with FFPE specimens is particularly valuable in lymphoma diagnostics, where complex rearrangements such as fusions involving *CCND2*, T-cell receptor (*TCR*), and immunoglobulin (*IG*) heavy and light chains are challenging to detect due to the limited availability of targeted FISH probes and the absence of fusion/chimeric transcripts detectable by RNA sequencing (Figure 4E). Similarly to RNA sequencing, GPM can detect gene fusions at exon-level resolution and can identify atypical fusions that may be missed by RT-PCR, such as the *BCR::ABL1* fusion involving *BCR* exon 13 and *ABL1* exon 3, as observed in case 8. Of note, the resolution of GPM has been evaluated in the constitutional genetics setting, which was comparable to OGM [15].

The limit of detection of GPM for mosaicism was >20% for deletions, duplications, and aneuploidy, and >30% for cnLOH based on data obtained from germline testing [15]. In this study, the in silico mixing test demonstrated a limit of detection of 10% for translocations and inversions, whereas CNAs (deletions, duplications, etc.) and cnLOH were not directly evaluated. Future work to fully define detection sensitivity across all variant types will include comprehensive, variant-specific dilution studies, ideally leveraging paired abnormal and remission samples from the same patient.

In most of the 18 cases, GPM demonstrated superior performance in detecting SVs and CNAs. The overall concordance for SV detection between GPM and routine cytogenomic methods was 93.5%, and for CNAs and cnLOH was 89.2%. One translocation (case 8) was not identified by GPM, possibly due to the relatively small size of the clone (~10%) around the detection threshold. One inversion of chromosome 7 (case 12) was not detected by GPM. A plausible explanation is the proportion of abnormal cells harboring inv(7) was lower in uncultured cells and fell below the detection threshold (10%) for GPM, whereas the clone was over-represented in cultured metaphase cells (20%) by karyotyping, likely due to culture bias that can occur in myeloid neoplasms. One *RUNX1* deletion (case 14) was not recognized by GPM, likely due to its small size placing it below the reliable detection threshold. One aneuploidy, present at a low level below the limit of detection, was also not identified. GPM failed to detect seven cnLOH (31.8%) that had been recognized by CMA, most likely due to low-level clones and the small size of cnLOH. Other limitations of GPM include relatively low sensitivity for detecting small inversions, particularly those that do not significantly disrupt local chromatin architecture or generate detectable proximity signals. These subtle SVs may go undetected if they fall below the resolution threshold of the assay or occur in regions with low contact frequency. The sensitivity of detecting cnLOH and small SVs may be improved by (1) increased sequencing depth to enhance signal-to-noise ratio and provide more Hi-C contact per locus, and (2) algorithmic refinements to improve normalization, noise modeling, and/or breakpoint-calling algorithms.

Additionally, GPM detected 62 SVs that were not identified by other conventional cytogenomic methods used in the routine clinical testing, demonstrating its potential to enhance sensitivity and to identify novel findings that may have clinical implications. Because of the retrospective nature of the study, a subset of the cases lacked a genome-wide genomic testing for comparison or were not feasible for additional confirmatory testing, limiting the ability to verify these variants and to assess the sensitivity of detection across all variant classes. Future clinical validation including orthogonal confirmation of novel or clinically actionable findings is warranted to demonstrate its performance and clinical utility.

### 4.2. GPM Improved Diagnostic Accuracy in Mature B-Cell and T-Cell Lymphomas

More genetic aberrations, particularly gene fusions, have been incorporated into diagnostic criteria as defining features for specific disease entities in the updated WHO and the ICC Classification of hematolymphoid neoplasms [1,2]. Accurate identification of these genetic drivers is critical for diagnosis and classification. Our data demonstrate that GPM confirms aberrations identified by routine cytogenomic methods, clarifies ambiguous chromosomal analysis and FISH results, and identifies additional clinically significant fusions and partners that were not detected by routine methods due to various limitations. While karyotyping and CMA are not routinely used in tissue biopsies for lymphoma, GPM offers advantages in detecting SVs and CNAs in FFPE samples.

In B-cell and T-cell lymphomas, chromosomal translocations frequently involve juxtaposition of genes under *IG* or *TCR* promoters, without the formation of fusion/chimeric transcripts. Unlike RNA sequencing, which only detects expressed fusion transcripts, GPM can identify gene juxtapositions regardless of whether they result in fusion transcript formation and provide a more comprehensive genomic analysis. FISH has limited target coverage, particularly for detecting *IGK* or *IGL* rearrangements, for which specific clinical testing is not routinely offered despite the availability of commerical probes, and it may misinterpret complex fusions. By accurately identifying both rearrangement partners, GPM facilitates definitive diagnostic classification. As illustrated in case 2, the detection of *IGL::MYC* by GPM provides further support to a diagnosis of Burkitt lymphoma, originally favored as high-grade B-cell lymphoma due to cytomorphologic variation and the absence of *IGH::MYC*. The exact frequency of *IGK::MYC* or *IGL::MYC* fusions in high-grade B-cell lymphomas, including Burkitt lymphoma, remains uncertain due to limited clinical testing for *IG* light chains. In a case with an original diagnosis as DLBCL transformed from FL (case 5), GPM offered distinct advantages by simultaneously identifying *PAX5::MYC* and *IGH::BCL2* rearrangements, along with additional CNAs including 11q aberrations and *TP53* deletion. This comprehensive genomic profiling integrated the disparate findings of FISH, RNA sequencing, and CMA, which had only partially captured the complexity of the genomic landscape. *PAX5* is a transcription factor that regulates B-cell development and modulates *MYC* expression through binding to the *MYC* promoter [24]. *PAX5::MYC* fusion has been reported in high-grade B-cell lymphoma with *MYC* and *BCL2* rearrangements, DLBCL, and transformed FL [25,26,27]. The integration of these genomic findings supports the classification as DLBCL/high-grade B-cell lymphoma with *MYC* and *BCL2* rearrangements. MYC expression is heterogeneous in cases with non-*IG::MYC* fusions [26], as observed in this case.

*CCND2* and *CCND3* rearrangements detected in most cyclin D1-negative mantle cell lymphoma represent a recurrent disease mechanism. These events are frequently mediated by cryptic repositioning of *IG* light chain enhancers/promoters juxtaposed to *CCND2* or *CCND3* (enhancer/promotor hijacking), resulting in aberrant overexpression of these cyclins [28]. Identification of an *IGL::CCND2* rearrangement by GPM demonstrates the diagnostic utility of this approach in clarifying cases that would otherwise remain diagnostically ambiguous by immunohistochemistry, conventional karyotyping, or FISH, as seen in case 4. Although the original diagnosis in this patient was CD5-positive DLBCL presumably transformed from the patient’s reported CLL/SLL, the presence of *IGL::CCND2* raises the possibility of mantle cell lymphoma at initial diagnosis. In addition, the concurrent identification of inv(3) *RPN1::EVI1* (*MECOM*), which corresponded to add(3) in a separate clone, highlights the ability of GPM to uncover additional structural variants across distinct clonal populations. Although well recognized as a recurrent abnormality in AML and other myeloid neoplasms [1,2], the bone marrow in case 4 showed no morphologic evidence of a myeloid neoplasm was identified. Therefore, the detection of *RPN1::EVI1* raises questions regarding its biological and clinical significance, an area that needs further investigation.

Establishing a definitive diagnosis of mature T-cell leukemias and lymphomas can be challenging when the immunophenotype is not specific and conventional testing is equivocal. In this setting, GPM showed clear diagnostic value by resolving the nature of a *TCL1A* abnormality that remained inconclusive by FISH, confirming a *TRA/D::TCL1A* fusion. The presence of a *TCL1A* rearrangement fulfills one of the essential diagnostic criteria for T-PLL, and can establish the diagnosis when integrated with clinical, morphologic, and immunophenotypic findings.

### 4.3. Diagnostic Relevance of GPM in B- and T-Lymphoblastic Leukemia/Lymphoma

The identification of *IGH::IL3* fusions [29,30] in B-LL further proved the unique capacity of GPM to detect promoter/enhancer juxtapositions that cannot be captured by RNA sequencing, as noted in mature lymphomas. B-LL with *IGH::IL3* fusion is a rare, distinct entity described in the revised 4th Edition WHO Classification and retained in the 5th Edition WHO and the ICC [1,2,31]. The resulting IL3 overexpression drives leukemogenesis and induces maturation and release of eosinophils from the bone marrow into peripheral blood [32,33]. Recognition of this rare entity is particularly important in patients with asymptomatic eosinophilia or hypereosinophilic syndrome to guide comprehensive genomic testing for accurate diagnosis.

*BCL11B* rearrangements define a heterogeneous group of acute leukemias that may present as AML, mixed phenotype acute leukemia (MPAL), T/myeloid, or T-LL [23]. These leukemias exhibit a distinct expression profile compared with other acute leukemia subtypes, and most rearrangements juxtapose *BCL11B* to super-enhancers, resulting in BCL11B overexpression that serves as a biomarker at diagnosis [23,34]. In our cohort, GPM identified a *BCL11B* rearrangement with an unknown partner gene in a T-LL with complex karyotype (case 11), an alteration that was not detected by conventional methods. The absence of detectable fusion transcripts by RNA sequencing and the lack of specific FISH probes for *BCL11B* emphasize the unique capacity of GPM to resolve such challenging genomic events. In addition, GPM detected biallelic *CDKN2A* deletion, but was not revealed by chromosomal analysis, although it was concordant with FISH. Deletions of *CDKN2A* are frequently observed in T-LL [35], detected at a frequency of about 30% by conventional karyotyping and at a higher frequency by molecular testing. These findings demonstrate the utility of GPM in uncovering both structural rearrangements and cryptic deletions, expanding diagnostic resolution beyond that of routine testing.

## 5. Conclusions

In summary, our data from this retrospective cohort demonstrate that GPM can precisely detect all types of SVs and CNAs in a single assay with sequence-level resolution, using fresh, frozen, and FFPE tissue, supporting its potential clinical utility in the diagnosis and accurate classification of hematologic neoplasms, particularly lymphomas. Our results showed that GPM detected both gene juxtapositions and chimeric gene fusions resulting from balanced and unbalanced chromosomal rearrangements, with 95.2% concordance with previously applied methods. As a whole-genome assay, GPM provides a powerful tool for detecting clinically actionable variants and uncovering novel aberrations that have not been previously identified. In some cases, these findings revealed driver mutations crucial for specific diagnoses and provided insights into disease pathogenesis. GPM offers the advantage of detecting CNAs and copy-neutral loss of heterozygosity (cnLOH) simultaneously in a single assay. Although our cohort is small and comprises selected diagnostically challenging cases, this study provides proof-of-concept evidence that the GPM assay has the potential to improve diagnostic accuracy in hematologic neoplasms and reduce the need for reflex testing. However, broader perspective studies in unselected patient populations will be necessary to validate its performance and clinical utility and to define its role in standard clinical testing algorithms.

## Data Availability

The original contributions presented in this study are included in the article. Further inquiries can be directed to the corresponding authors.

## Abbreviations

The following abbreviations are used in this manuscript:

AML	Acute myeloid leukemia
B-LL	B-lymphoblastic leukemia/lymphoma
CBD	Contact by discontinuity
CLL/SLL	Chronic lymphocytic leukemia/small lymphocytic lymphoma
CMA	Chromosomal microarray analysis
CML	Chronic myeloid leukemia
CNA	Copy number alteration
cnLOH	Copy-neutral loss of heterozygosity
DLBCL	Diffuse large B-cell lymphoma
FFPE	Formalin-fixed, paraffin-embedded
FISH	Fluorescence in situ hybridization
FL	Follicular lymphoma
GPM	Genomic proximity mapping
Hi-C	High-throughput chromosome conformation capture
ICC	International Consensus Classification
IG	Immunoglobulin
LRS	Long-red sequencing
MDS	Myelodysplastic neoplasm
MPAL	Mixed phenotype acute leukemia
NGS	Next-generation sequencing
OGM	Optical genome mapping
PMF	Primary myelofibrosis
SV	Structural variant
TCR	T-cell receptor
T-LL	T-lymphoblastic leukemia/lymphoma
T-PLL	T-prolymphocytic leukemia
CNN	Convolutional neural network
MAF	Minor allele frequency
